# Supplementary material for: Cytokine Expression Patterns and Single Nucleotide Polymorphisms (SNPs) in Patients with Chronic Borreliosis
Source: Antibiotics (Basel). 2019 Jul 30;8(3):107. doi: 10.3390/antibiotics8030107 (PMC6784230; doi:10.3390/antibiotics8030107)
Supplement: Supplementary file 1 [file antibiotics-08-00107-s001.pdf]

antibiotics

MDPI

Supplementary Materials

Cytokine Expression Patterns and Single Nucleotide Polymorphisms (SNPs) in Patients with Chronic Borreliosis

Tabea M. Hein [1], Philip Sander [1], Anwar Giryes [2], Jan-Olaf Reinhardt [3], Josef Hoegel [4] and E. Marion Schneider [1,*]

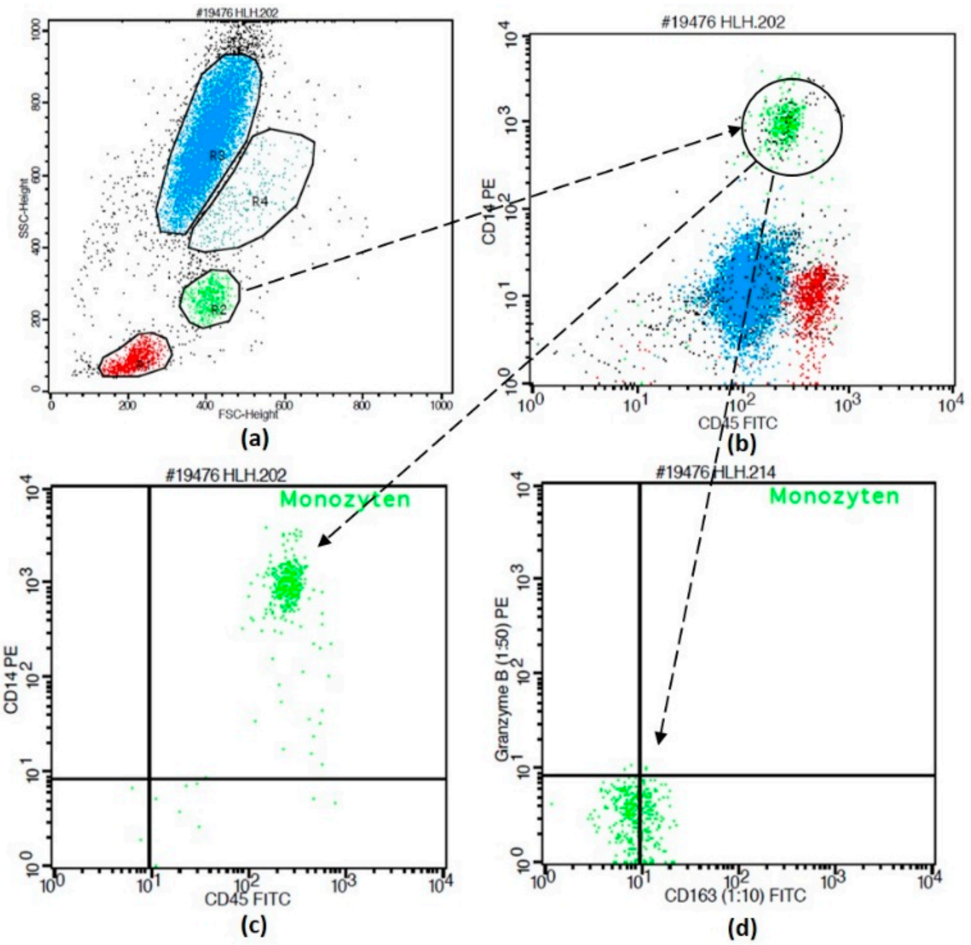

Figure S1. Gating Strategy for the Identification of CD163+ Monocytes. (a) Scatter plot with the different leukocyte populations: Lymphocyte gate R1 (red dots), monocyte gate R2 (green dots), two granulocyte gates R3 (blue) and R4 (light blue). Forward Scatter (FSC-Height; x-axis), Side Scatter (SSC-Height; y-axis). (b) Dot plot identifying leukocytes (CD45-FITC positive, x-axis), and monocytes (CD45-FITC positive and CD14-PE positive, y-axis). (c) Dot plot showing $CD45^{+}CD14^{+}$ monocytes. (d) Dot plot CD163-FITC expression by monocytes (x-axis), being Granzyme B-PE negative (y-axis).

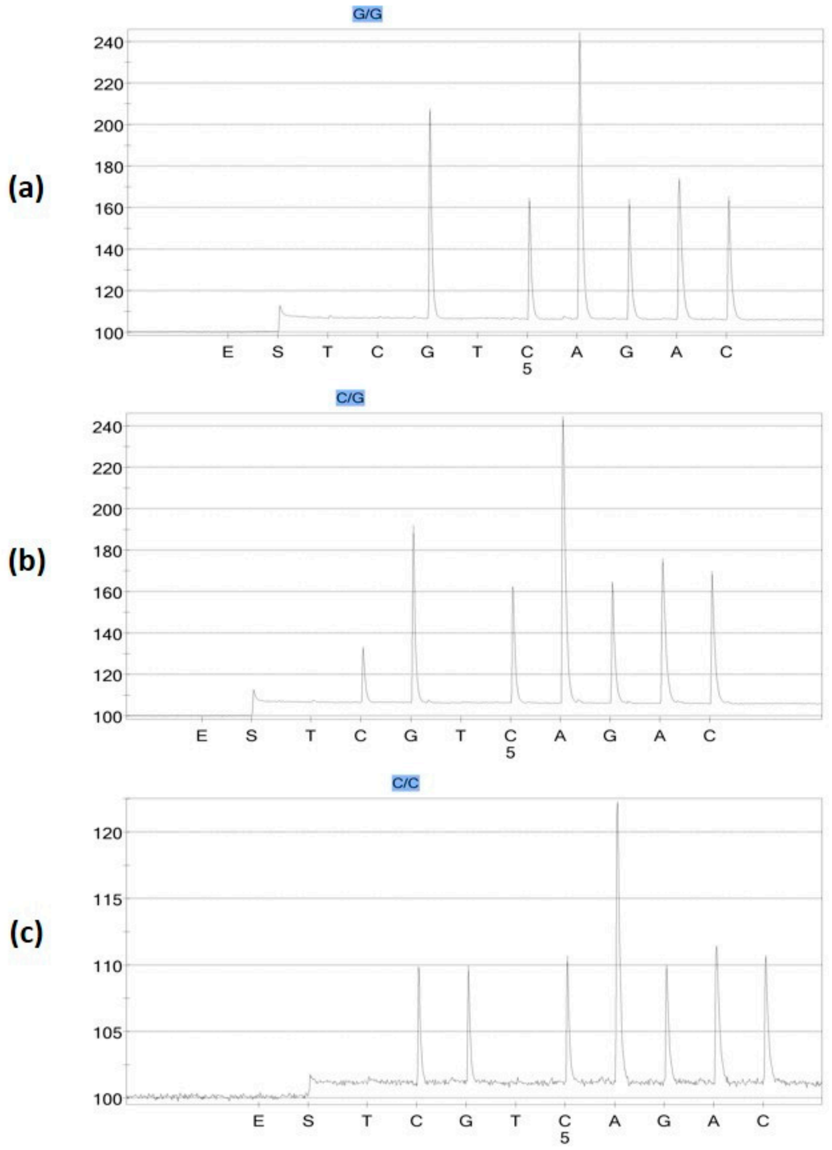

Figure S1. Representative Pyrograms of IL-6 Promotor SNP rs1800795 Genotypes. (a) homozygous C/C genotype. (b) heterozygous C/G genotype. (c) homozygous G/G genotype. SNPs have been sequenced in reverse. X-Axis indicates the sequencing order of the nucleotides (E – enzyme mix, S – substrate mix, GCTA – nucleotides) , y-axis the intensities of respective luminescent signals correlation to the number of nucleotides inserted. The second nucleotide is the polymorphic position.
